# Non‐Fermi Liquids as Highly Active Oxygen Evolution Reaction Catalysts

**DOI:** 10.1002/advs.201700176

**Published:** 2017-06-06

**Authors:** Shigeto Hirai, Shunsuke Yagi, Wei‐Tin Chen, Fang‐Cheng Chou, Noriyasu Okazaki, Tomoya Ohno, Hisao Suzuki, Takeshi Matsuda

**Affiliations:** ^1^ Department of Materials Science and Engineering Kitami Institute of Technology 165 Koen‐cho Kitami 090‐8507 Japan; ^2^ Institute of Industrial Science The University of Tokyo 4‐6‐1 Komaba Meguro‐ku Tokyo 153‐8505 Japan; ^3^ Center for Condensed Matter Sciences National Taiwan University Taipei 10617 Taiwan; ^4^ Taiwan Consortium of Emergent Crystalline Materials Ministry of Science and Technology Taipei 10622 Taiwan; ^5^ National Synchrotron Radiation Research Center Hsinchu 30076 Taiwan; ^6^ Department of Biotechnology and Environmental Chemistry Kitami Institute of Technology 165 Koen‐cho Kitami 090‐8507 Japan; ^7^ Research Institute of Electronics Shizuoka University 3‐5‐1 Johoku Hamamatsu Shizuoka 432‐8011 Japan

**Keywords:** non‐Fermi liquids, overpotential, oxygen evolution reaction, transition metal oxides

## Abstract

The oxygen evolution reaction (OER) plays a key role in emerging energy conversion technologies such as rechargeable metal‐air batteries, and direct solar water splitting. Herein, a remarkably low overpotential of ≈150 mV at 10 mA cm^−2^_disk_ in alkaline solutions using one of the non‐Fermi liquids, Hg_2_Ru_2_O_7_, is reported. Hg_2_Ru_2_O_7_ displays a rapid increase in current density and excellent durability as an OER catalyst. This outstanding catalytic performance is realized through the coexistence of localized d‐bands with the metallic state that is unique to non‐Fermi liquids. The findings indicate that non‐Fermi liquids could greatly improve the design of highly active OER catalysts.

The complex oxygen evolution reaction (OER) is the crux of emerging energy conversion technologies like metal‐air batteries^1^, ^2^ and direct solar water splitting.^3^, ^4^ Although catalysts based on perovskites,^5^, ^6^, ^7^, ^8^, ^9^ oxy‐hydroxides,^10^, ^11^ RuO_2_ and IrO_2_ 12 display high current densities and facilitate the reaction, most of them have room for improvement in their overpotentials and conversion efficiencies. Here, a remarkably low overpotential of ≈150 mV at 10 mA cm^−2^_disk_ in alkaline solutions is reported for OER using one of the non‐Fermi liquids (with anomalous metallic state caused by strong electron–electron interactions), Hg_2_Ru_2_O_7_. Hg_2_Ru_2_O_7_ displays a rapid increase in the current density and an excellent durability as an OER catalyst. This outstanding catalytic performance is realized through the coexistence of localized d‐bands with the metallic state of the non‐Fermi liquid. These findings indicate that non‐Fermi liquids could greatly improve the design of highly active OER catalysts.

Recent reports^5^, ^6^, ^7^, ^8^, ^9^, ^10^ on bulk electronic states of transition metal ions manifesting on the catalyst surface have greatly advanced our understanding of OER catalysts. Through their evaluation of OER activities on insulating AMO_3_ perovskite oxides, Suntivich et al.^5^ suggested that catalysts exhibit maximum OER activity when the number of d‐electrons in the e_g_ orbital was near unity for transition metal ions occupying the octahedral sites.^5^ Since the σ‐bonding e_g_ orbital has stronger overlap with adjacent O 2p orbitals of oxygen adsorbates, electron transfer between the M‐cation and OER intermediates is faster. For insulating AMO_3_ perovskite oxides, the unoccupied e_g_ level in the 3d conduction band is lowered by the applied overpotential (η) in order to overlap with the O 2p band of oxygen adsorbates (**Figure** **1**a). The gap (Δ*_η_*) between the O 2p valence band and the unoccupied 3d conduction band can then be treated as a measure of overpotential, or as a direct proxy of the OER activity. This gap is caused by the on‐site Coulomb potential *U*_dd_ (Figure 1) that localizes the d‐bands. When the gap is small, the overpotential and corresponding Tafel slope^13^, ^14^ are also small, leading to rapid electron transfer between the M‐cation and the oxygen adsorbates. In general, insulating perovskite oxides have high OER activity, but require high overpotentials due to the wide gap between the O 2p valence band and the unoccupied 3d conduction band. One solution is to design a catalyst composed of several transition metals with multiple OER active sites. As for gelled FeCoW oxy‐hydroxide_,_ 10 such a design can sometimes greatly enhance the catalytic activity through synergistic interplay between transition metals. However, the large gap between the O 2p valence band and the unoccupied 3d conduction band still leads to a substantial overpotential.

**Figure 1:**
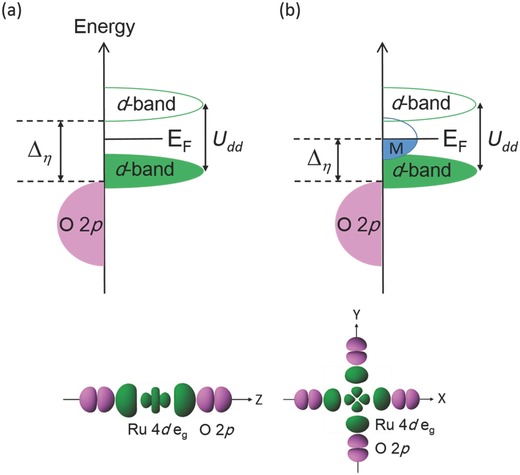
Schematic representation of the electronic structure in transition metal oxides. The electronic structure of a) a Mott–Hubbard insulator (corresponds to the insulating phase of Hg_2_Ru_2_O_7_) and b) a non‐Fermi liquid where the localized d‐band coexists with the metallic state (corresponds to the metallic phase of Hg_2_Ru_2_O_7_). The electronic structure of Hg_2_Ru_2_O_7_ is based on that of Chainani et al.,^22^ who showed that the metallic state is merely composed of Ru 4d‐electrons. E_F_ and M denote the Fermi level and the metallic state of d‐electrons, respectively. Δ*_η_* is a descriptor of the overpotential necessary for the unoccupied d‐level to overlap with the O p‐level of the oxygen adsorbates. For a charge‐transfer insulator, the positions of the occupied d‐band of the transition metal oxide and the O 2p band of the oxygen adsorbates are switched in (a).

A separate approach could employ metallic oxides with no d–d gap. Due to their metallic state, the gap between the O 2p valence band and Fermi level (E_F_) corresponds to the overpotential. Since this gap is smaller compared with that in insulators, a smaller overpotential and faster electron transfer would be expected. In this case, overlap between the unoccupied metallic state (where antibonding e_g_ orbitals exist) and the O 2p band of the oxygen adsorbates becomes too large, and rapid amorphization occurs at the surface during electron transfer when bonds are weakened.^8^, ^15^, ^16^

A separate compromise would be the use of a material where a metallic state of d‐electrons exists between the localized d‐bands. In such a material, the metallic state, but not the localized and unoccupied d‐band, would overlap with the O 2p band of the oxygen adsorbates (Figure 1b). Therefore, overlap between antibonding e_g_ orbitals and O 2p orbitals only occurs in the metallic state, which preserves the bond strength during electron transfer.

This work proposes a non‐Fermi liquid^17^, ^18^ that satisfies the criterion for coexistence of localized d‐bands and the metallic state (Figure 1b). Specifically, the OER activity of the room‐temperature non‐Fermi liquid Hg_2_Ru_2_O_7_ 19, 20, 21, 22, 23 was evaluated in detail. Hg_2_Ru_2_O_7_ (Ru^5+^: 4d, t_2g_^3^e_g_^0^) undergoes a metal–insulator transition at 108 K.^19^, ^20^, ^21^ However, the localized d‐bands of the Mott–Hubbard^24^ insulator (Figure 1a) are retained across the transition^22^ and the system adopts a non‐Fermi liquid state above 108 K^22^, ^23^ (Figure 1b). Non‐Fermi liquid systems are d‐ or f‐electron systems characterized by strong electron–electron interactions that prevent entry into the Fermi liquid ground state^18^ and some of them exhibit superconductivity.^25^, ^26^ The fascination with the physics of non‐Fermi liquids is the unusual temperature dependence of their physical properties.^17^, ^18^, ^27^ For example, the electrical resistivity (ρ) of Hg_2_Ru_2_O_7_ increases proportionally to *T* above 108 K,^19^, ^20^, ^21^, ^23^ instead of *T* ^2^ as in Fermi liquids.

The catalytic OER activity of the non‐Fermi liquid Hg_2_Ru_2_O_7_ was studied in comparison with Ca_2_Ru_2_O_7_,^28^ Cd_2_Ru_2_O_7_,^29^ and RuO_2_ on glassy carbon disk electrodes. The OER measurements were conducted in 0.1 m KOH aqueous solutions and the current densities were normalized to the glassy carbon disk electrode area. Hg_2_Ru_2_O_7_ was confirmed to be single‐phase with the pyrochlore structure (**Figure** **2**) by synchrotron X‐ray diffraction (see the XRD profile in Figure S1, Supporting Information). Also, the Ru^5+^ valence in this compound was confirmed by X‐ray photoemission spectra (see the Ru 3d_5/2_ peak in Figure S8, Supporting Information). Hg_2_Ru_2_O_7_ particles are in the size of 1–5 µm (see the scanning electron microscopy (SEM) image in Figure S2, Supporting Information).

**Figure 2:**
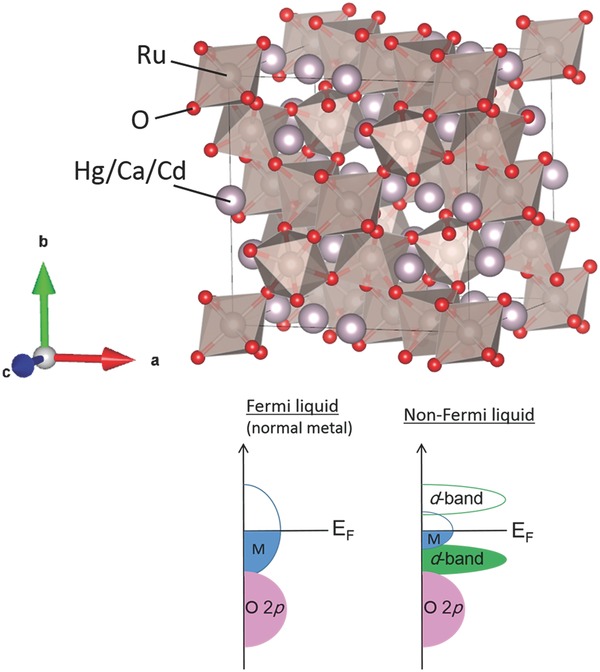
Schematic representation of the crystal structure of the cubic pyrochlore Hg_2_Ru_2_O_7_ (Ca_2_Ru_2_O_7_ and Cd_2_Ru_2_O_7_ are isostructural with Hg_2_Ru_2_O_7_). The A‐site cation has an eightfold oxygen coordination, while Ru has a sixfold oxygen coordination. Both A‐ and B‐site cations independently form corner‐sharing tetrahedral networks. Hg_2_Ru_2_O_7_ adopts a non‐Fermi liquid state above 108 K.^22^, ^23^

**Figure** **3**a shows the capacitance‐corrected voltammograms for the isostructural Hg_2_Ru_2_O_7_, Ca_2_Ru_2_O_7_, and Cd_2_Ru_2_O_7_. The OER activity of Hg_2_Ru_2_O_7_ is significantly larger than other cubic pyrochlore ruthenates. At an applied potential of 1.5 V versus RHE a current density of ≈100 mA cm^−2^ was recorded for Hg_2_Ru_2_O_7_ (Figure 3b), which is comparable to gelled nanoporous FeCoW oxy‐hydroxide (we note that the mass loading of FeCoW oxy‐hydroxide is much larger than Hg_2_Ru_2_O_7_).^10^ Since high OER activity was also reported for Pb_2_Ru_2_O_6.5_ and Bi_2.4_Ru_1.6_O_7_ pyrochlore oxides by Parrondo et al.,^30^ catalysts with pyrochlore structure are potentially highly active OER catalysts apart from the well‐known perovskite oxides.^5^, ^6^ The mass OER activity of Hg_2_Ru_2_O_7_ at 1.5 V versus RHE is ≈400 mA mg^−1^, while that of Pb_2_Ru_2_O_6.5_ is ≈200 mA mg^−1^.^30^ Figure 3c–e shows the *iR*‐corrected Tafel plots obtained under steady‐state conditions. All tested cubic pyrochlore ruthenates exhibited two Tafel slopes (Figure 3c–e). The two clear Tafel slopes in Figure 3c–e may suggest a similar switch in the rate‐determining step for OER at ≈1.52 V as noted for RuO_2_.^31^ However, the absolute value of the Tafel slope was not used to determine the reaction step (in the OER) due to the delicate use of the Tafel equation as pointed out by Gileadi and Kirowa‐Eisner.^32^ The smaller Tafel slope in Figure 3c together with the lower onset potential demonstrates that Hg_2_Ru_2_O_7_ exhibits higher OER activity than other cubic pyrochlore ruthenates. Although the temperature dependence of the electrical resistivity of Ca_2_Ru_2_O_7_ (Ru^5+^: 4d, t_2g_^3^e_g_^0^) and Cd_2_Ru_2_O_7_ (Ru^5+^: 4d, t_2g_^3^e_g_^0^) resembles an insulator (the d‐bands are gapped), their electrical resistivity at room temperature (2–5 mΩ cm) is comparable to metals.^28^, ^29^ As stated by Miyazaki et al.,^33^ the metal–insulator transition of Ca_2_Ru_2_O_7_ and Cd_2_Ru_2_O_7_ is suppressed to a small anomaly at ≈25 and ≈30 K in sharp contrast with Hg_2_Ru_2_O_7_. This suggests that the metallic states in Ca_2_Ru_2_O_7_ and Cd_2_Ru_2_O_7_ are suppressed in comparison with Hg_2_Ru_2_O_7_. Since the electronic bands in Figure S7 (Supporting Information) are composed of Ru 4d‐electrons, the localized d‐bands of Ca_2_Ru_2_O_7_ and Cd_2_Ru_2_O_7_ become wider due to the narrower metallic states. Accordingly, the reason why Cd_2_Ru_2_O_7_ (Figure 3e) has a lower OER activity than Ca_2_Ru_2_O_7_ is likely to be the wider d‐band and the narrower metallic state (Figure S7, Supporting Information). **Figure** **4** compares the overpotential of Hg_2_Ru_2_O_7_, Ca_2_Ru_2_O_7_, Cd_2_Ru_2_O_7_, and RuO_2_ with other highly active oxide OER catalysts.^5^, ^6^, ^9^, ^10^, ^30^, ^34^ Here, the overpotential of Hg_2_Ru_2_O_7_ (η = ≈150 mV at 10 mA cm^−2^_disk_) is superior to other highly active OER catalysts (e.g., NiFeOOH^34^) and it is the lowest OER overpotential for oxide catalysts in alkaline solutions reported to date. Also, Hg_2_Ru_2_O_7_ outperforms among the Ru‐based catalysts, showing the lowest overpotential and the highest TOF (see the OER activity in Table S6, Supporting Information). Furthermore, the overpotential of Hg_2_Ru_2_O_7_ is 174(8) mV at 20 mA cm^−2^ and 269(16) mV at 100 mA cm^−2^, exhibiting the comparable values with state‐of‐the‐art OER catalysts including oxygen‐incorporated Ni—Fe—S^35^ (259 mV at 20 mA cm^−2^) and NiFe_2_O_4_—NiOOH^36^ (270 mV at 100 mA cm^−2^). The low overpotential and a small Tafel slope can be rationalized in terms of the electronic structure of Hg_2_Ru_2_O_7_ (Ru^5+^: 4d, t_2g_^3^e_g_^0^) where the localized d‐band coexists with the metallic state (Figure 1b). Here, the Hg 5d states (binding energy: 6–12 eV) do not contribute to OER since their d‐levels are distributed far away from the Fermi level.^22^ Also, any correlation between structural differences and the catalytic activity can be neglected since the bond distances and bond angles of the cubic pyrochlore ruthenates are almost identical (see the crystallographic data in Table S1, Supporting Information). Since the metallic state exists between the localized d‐bands, the unoccupied e_g_ level at the Fermi level is lowered by the overpotential and overlaps with the O 2p band of the oxygen adsorbates (Figure 1b). In this way, the electron transfer between the Ru e_g_ orbital and the O 2p orbital of the oxygen adsorbates becomes rapid. In summary, all these figures of electrochemical properties (Figures 3 and 4) demonstrate that the OER performance of Hg_2_Ru_2_O_7_ outperforms among the existing oxide OER catalysts.

**Figure 3:**
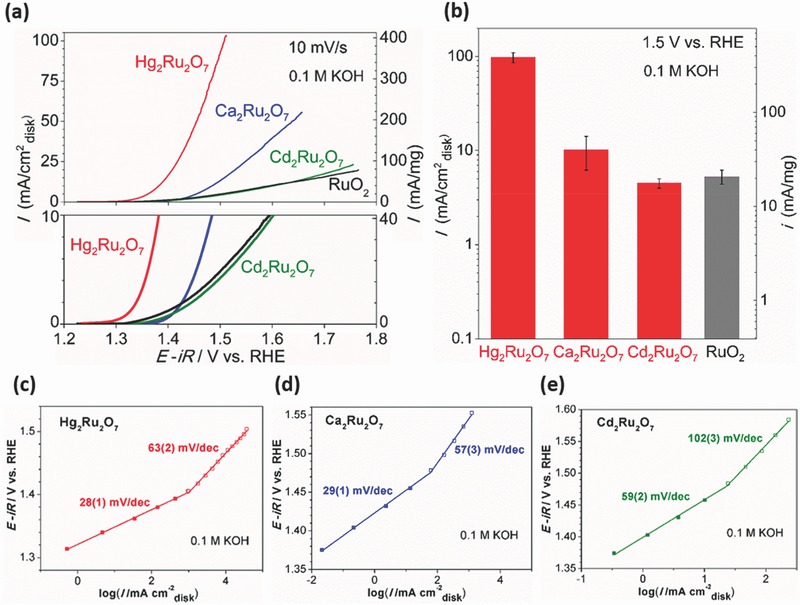
OER catalytic activity of the cubic pyrochlore ruthenates. a) Linear sweep voltammograms at the scan rate of 10 mV s^−1^ for Hg_2_Ru_2_O_7_, Ca_2_Ru_2_O_7_, and Cd_2_Ru_2_O_7_. Cycle 10 was used as a representative for each catalyst. The OER measurements were conducted in 0.1 m KOH solutions. The averaged linear sweep voltammograms instead of the cyclic voltammograms are shown here due to the negligible double‐layer capacitance in this scale. The current density *I* was both normalized to the glassy carbon disk electrode area (0.15 × 0.15 × π cm^2^) and the catalytic mass (0.018 mg). Hg_2_Ru_2_O_7_ reaches the current density of 10 mA cm^−2^_disk_ at 1.378 V versus RHE for cycle 10. The linear sweep voltammetry curve of RuO_2_ (this study) is shown as a reference. b) The OER current density (mA cm^−2^_disk_ and mA mg^−1^) at 1.5 V versus RHE for Hg_2_Ru_2_O_7_ (cycle 1–100), Ca_2_Ru_2_O_7_ (cycle 1–50), Cd_2_Ru_2_O_7_ (cycle 1–50), and RuO_2_ (cycle 1–50). Tafel plots for c) Hg_2_Ru_2_O_7_, d) Ca_2_Ru_2_O_7_, and e) Cd_2_Ru_2_O_7_, collected under steady‐state conditions. Standard deviation for the data is within the symbol size.

**Figure 4:**
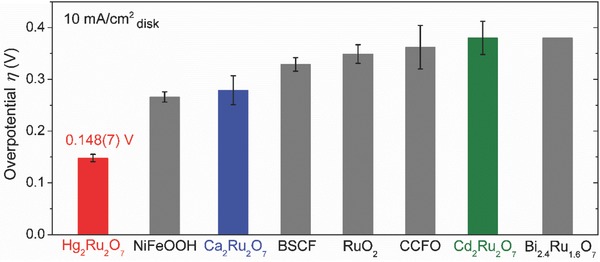
OER overpotentials of cubic pyrochlore ruthenates and other highly active oxide (and oxy‐hydroxide) OER catalysts. The overpotential (η) at the geometric current density of 10 mA cm^−2^_disk_ was determined by η = *E* − 1.23 V. All the catalysts here have small Brunauer–Emmett–Teller (BET) surface areas, and their current densities were normalized to the glassy carbon disk electrode area instead of the BET surface area. Since OER measurements for these catalysts were conducted in KOH solutions, the overpotentials are compared to those of NiFeOOH (Batchellor and Boettcher^34^), BSCF (Suntivich et al.^5^), RuO_2_ (this study), CCFO (Yagi et al.^6^), and Bi_2.4_Ru_1.6_O_7_ (Parrondo et al.^30^) under controlled conditions.

Extended electrochemical studies were conducted to investigate the catalytic stability of Hg_2_Ru_2_O_7_. The chronoamperometric curve in **Figure** **5**a shows that the OER activity of Hg_2_Ru_2_O_7_ is stable in 0.1 m KOH with the current density (at a constant voltage of 1.5 V vs RHE) sustaining ≈95% of the initial value even after 100 h, while the current density of Ca_2_Ru_2_O_7_ decreases down to ≈59% of the initial value after 100 h. The catalytic stability of Hg_2_Ru_2_O_7_ was also tested in 1.0 and 6.0 m KOH electrolyte, exhibiting excellent stability of ≈95% regardless of the KOH molarity (Figure S11, Supporting Information). Figure 5b shows the voltammograms of Hg_2_Ru_2_O_7_ and Ca_2_Ru_2_O_7_ over 50–100 cycles. The current density of Ca_2_Ru_2_O_7_ decreases with cycle number, while that of Hg_2_Ru_2_O_7_ increased due to an increase in surface area by the slight progression of amorphization. Therefore, Figure 5a,b demonstrates that Hg_2_Ru_2_O_7_ possesses an OER stability comparable to the very stable CaCu_3_Fe_4_O_12_.^6^ The stability of Hg_2_Ru_2_O_7_ was further probed using high‐resolution transmission electron microscopy (HRTEM) both before and after 50 or 100 OER cycles. Figure 5c,d shows the HRTEM and fast Fourier transform (FFT) images of Hg_2_Ru_2_O_7_ and Ca_2_Ru_2_O_7_ before and after OER measurements. Before getting into detail about the catalytic stability, we note that Hg_2_Ru_2_O_7_ is a non‐Fermi liquid, while Ca_2_Ru_2_O_7_ is in the vicinity of a metal and an insulator. The electrical resistivity of Hg_2_Ru_2_O_7_ (at room temperature) is in the range of a metal,^19^, ^20^ and its temperature dependence is characteristic to non‐Fermi liquids.^19^, ^20^ On the other hand, the electrical resistivity of Ca_2_Ru_2_O_7_ (at room temperature) is in the range of a metal,^28^ while its temperature dependence resembles an insulator^28^ (suggesting the existence of a band gap). Also, as reported by Yagi et al.^6^ and May et al.,^37^ the catalytic activity of an OER catalyst is determined by the ≈10 nm layer of the catalytic surface. As‐synthesized, both Hg_2_Ru_2_O_7_ and Ca_2_Ru_2_O_7_ possess highly crystalline surface structures. However, after casting, ≈5 nm amorphous layer is immediately formed on their catalytic surface (Figure 5c,d). In other words, the OER measurement starts with a similar surface state for Hg_2_Ru_2_O_7_ and Ca_2_Ru_2_O_7_. However, there is a clear difference between Hg_2_Ru_2_O_7_ and Ca_2_Ru_2_O_7_ in their cycle dependence toward the OER measurement (Figure 5b). The current density for Hg_2_Ru_2_O_7_ gradually increases with cycling up to cycle 100, while that for Ca_2_Ru_2_O_7_ clearly decreases with cycling after cycle 10. This contrast can be attributed to the difference of the amorphous layer thickness after multiple cycling (Hg_2_Ru_2_O_7_: ≈5 nm after 100 cycles, Ca_2_Ru_2_O_7_: >25 nm after 50 cycles). As reported by May et al.,^37^ amorphization gradually increases the electrochemically active surface area of the catalyst. On the other hand, the electrical resistivity increases with amorphization (which leads to the decrease of the OER current density) due to the lattice irregularity of the amorphous layer. We note that the initial electronic bands (near the Fermi level) of the crystalline layer are maintained in the amorphous layer and the surface electronic structure is analogous to the bulk electronic structure. Therefore, the lattice irregularity of the amorphous layer not only increases the electrical resistivity, but it also leads to the increase of the band gap in the case of Ca_2_Ru_2_O_7_ due to its insulating character of the temperature dependence.^28^ In the case of Hg_2_Ru_2_O_7_, the growth of the amorphous layer is small even after 100 cycles (Figure 5c). The diffused Hg^2+^ provided by the crystalline layer thus reaches the outermost surface after 100 OER cycles due to the thin amorphous layer of ≈5 nm and finally dissolves in the alkaline solution (see the compositional information in Table S4 (Supporting Information) determined by the integrated XPS peak areas in Figure S9, Supporting Information). Therefore, the electrochemically active surface area slightly increases and the electrical resistivity does not increase due to the almost constant thickness (≈5 nm) of the amorphous layer (the thickness ratio between the crystalline layer and amorphous layer remains almost constant at the ≈10 nm catalytic surface). For this reason, the OER current density of Hg_2_Ru_2_O_7_ slightly increases with cycling. We also note that the dissolved Hg^2+^ in the alkaline solution (Table S4, Supporting Information) does not alter the initial electronic structure since the electronic bands (near the Fermi level) are merely composed of the Ru 4d‐electrons. In the case of Ca_2_Ru_2_O_7_, the growth of the amorphous layer was ≈20 nm (Figure 5d after 50 cycles), so the ≈10 nm catalytic surface is merely composed of the amorphous layer and the electrical resistivity increases rapidly, totally exceeding the increase in electrochemically active surface area. Therefore, the OER current density of Ca_2_Ru_2_O_7_ starts to decrease after cycle 10 and becomes less than half at 1.5 V versus RHE for cycle 50 (at cycle 10, the increase of the electrochemically active surface area cancels out the increase of the electrical resistivity). We note that the chemical analysis via XPS (Table S4, Supporting Information) suggests that the diffused Ca^2+^ continuously provided by the crystalline layer did not reach the outermost surface due to the thick amorphous layer of ≈25 nm. The competition between the increase in electrochemically active surface area and increase in the electrical resistivity was also described by Yagi et al.,^6^ comparing the stable CCFO after 100 OER cycles (with an amorphous layer of ≈5 nm corresponding to Hg_2_Ru_2_O_7_ in our study) with the unstable CFO after 100 OER cycles (with an amorphous layer of ≈20 nm corresponding to Ca_2_Ru_2_O_7_ in our study). The difference of the extent of amorphization in Hg_2_Ru_2_O_7_ and Ca_2_Ru_2_O_7_ during the OER can be explained by bonding strength as demonstrated by Yagi et al.^6^ Hg_2_Ru_2_O_7_ has a strong covalent bonding network (see the ionicity in Table S3, Supporting Information) formed by Hg—O and Ru—O (involving both A‐ and B‐sites in Figure 2). However, Ca_2_Ru_2_O_7_ possesses Ca—O ionic bonds (the A‐site in Figure 2) and Ru—O covalent bonds (the B‐site in Figure 2). In other words, Ca^2+^ is continuously provided by the crystalline layer increasing the thickness of the amorphous layer, while most of the Hg^2+^ and Ru^5+^ of Hg_2_Ru_2_O_7_ are retained in the crystalline layer, resulting in an almost constant thickness of the amorphous layer during OER. This explanation is based on the chemical analysis via XPS (see the compositional information in Table S4, Supporting Information) and it has newly revealed the source of A‐site cation diffusion, which has not been clarified since the findings of Yagi et al.^6^ In summary, Hg_2_Ru_2_O_7_ is both a highly active and stable OER catalyst.

**Figure 5:**
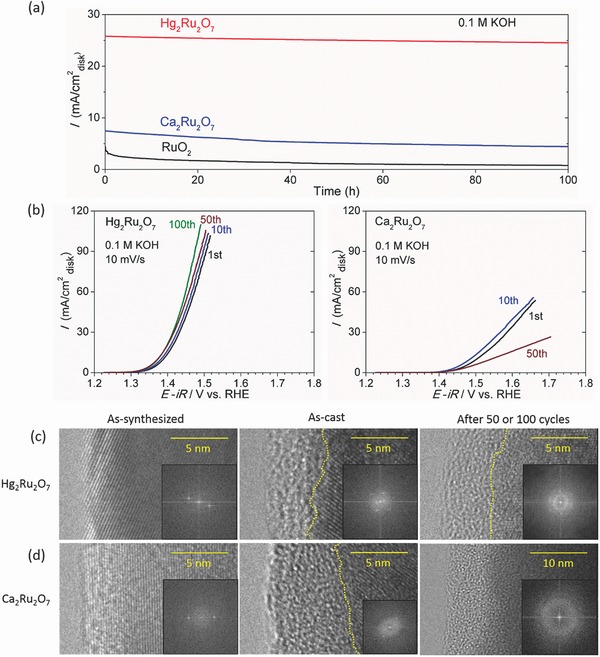
OER catalytic stability of the cubic pyrochlore ruthenates. a) Chronoamperometric curves at 1.5 V versus RHE (without *iR*‐correction) for Hg_2_Ru_2_O_7_ and Ca_2_Ru_2_O_7_ for 100 h in 0.1 m KOH solutions. The chronoamperometric curve at 1.5 V versus RHE (without *iR*‐correction) for RuO_2_ is shown as a reference. b) The cycle dependence of the linear sweep voltammograms at the scan rate of 10 mV s^−1^ for Hg_2_Ru_2_O_7_ (100 cycles) and Ca_2_Ru_2_O_7_ (50 cycles). The OER measurements were conducted in 0.1 m KOH solutions. HRTEM and fast Fourier transform (FFT) images before and after the OER measurements for c) Hg_2_Ru_2_O_7_ (100 cycles) and d) Ca_2_Ru_2_O_7_ (50 cycles). All the FFT images were obtained from surface regions of 10 × 10 nm^2^. The boundaries between the crystalline and amorphous regions are divided by yellow dotted lines. The lattice fringes in (c) correspond to (4 0 0) (*d* = 0.255 nm) for as‐synthesized, (3 1 1) (*d* = 0.308 nm) and (4 0 0) (*d* = 0.255 nm) for as‐cast, and (4 0 0) (*d* = 0.255 nm) for after 100 cycles. The lattice fringes in (d) correspond to (3 3 1) (*d* = 0.234 nm) for as‐synthesized and (4 0 0) (*d* = 0.255 nm) for as‐cast.

In conclusion, the non‐Fermi liquid Hg_2_Ru_2_O_7_ exhibited the lowest reported OER overpotential for oxide catalysts (≈150 mV at 10 mA cm^−2^_disk_) in alkaline solutions with a rapid increase in the current density and excellent catalytic stability. More importantly, this outstanding OER performance can be explained in terms of the coexistence of the localized d‐bands and the metallic state in the material. These findings indicate that the principles of non‐Fermi liquids could serve as new design criteria for highly active OER catalysts.

## Conflict of Interest

The authors declare no conflict of interest.

## Supporting information

Supplementary
