# Supplementary material for: Non‐Fermi Liquids as Highly Active Oxygen Evolution Reaction Catalysts
Source: Adv Sci (Weinh). 2017 Jun 6;4(10):1700176. doi: 10.1002/advs.201700176 (PMC5644224; doi:10.1002/advs.201700176)
Supplement: Supplementary file 1 — Supplementary [file ADVS-4-na-s001.pdf]

Copyright WILEY-VCH Verlag GmbH & Co. KGaA, 69469 Weinheim, Germany, 2017.

ADVANCED SCIENCE
Open Access

Supporting Information

for *Adv. Sci.,* DOI: 10.1002/advs.201700176

Non-Fermi Liquids as Highly Active Oxygen Evolution Reaction Catalysts

Shigeto Hirai, Shunsuke Yagi, Wei-Tin Chen, Fang-Cheng Chou, Noriyasu Okazaki, Tomoya Ohno, Hisao Suzuki, and Takeshi Matsuda*

Supporting Information

Non-Fermi Liquids as Highly Active Oxygen Evolution Reaction Catalysts

Shigeto Hirai*, Shunsuke Yagi, Wei-Tin Chen, Fang-Cheng Chou, Noriyasu Okazaki, Tomoya Ohno, Hisao Suzuki, and Takeshi Matsuda

I. Experimental Section

Sample synthesis

Polycrystalline samples of cubic pyrochlore ruthenates were prepared from a stoichiometric mixture of HgO (ACROS, 99 %), CaO (obtained from calcined $CaCO_3$, Alfa Aesar, 99.9965 %), CdO (Showa, 99.5 %), and RuO_2 (Alfa Aesar, 99.95 %). The powder was pressed with the oxidizer $KClO_4$ (J. T. Baker, 99.3 %) in a Au capsule and sintered at 950 °C for 1 h under 6 GPa using a cubic-anvil-type high-temperature and high-pressure apparatus (TRY Engineering Co., Japan). Both the suppression of HgO decomposition and the stabilization of Ru^{5+} under high oxygen pressure can be realized by high-pressure synthesis.

Characterization

Synchrotron X-ray powder diffraction profiles of cubic pyrochlore ruthenates were obtained at the BL01C2 beamline, National Synchrotron Radiation Research Center (NSRRC) with a wavelength of λ = 0.619925 Å. Crystal structure refinements of cubic pyrochlore ruthenates were conducted based on the obtained synchrotron X-ray powder diffraction data using Rietveld refinement software (Bruker AXS TOPAS [S1]).

Preparation of the catalyst inks

The catalyst inks for the electrochemical measurements were prepared referring to methods reported by Suntivich *et al.*[5] and Grimaud *et al.*[8]. K^+ ion-exchanged Nafion® was used as an immobilizing binder, enabling smooth transport of dissolved O_2 to the surface of the catalysts. A 3.33 wt.% K^+ ion-exchanged Nafion® suspension was first obtained from a mixture of 5 wt.% proton-type Nafion® suspension (Sigma-Aldrich) and a 0.1 M KOH aqueous solution in a 2:1 (*v/v*) ratio. This process increased the pH of the

initial 5 wt.% proton-type Nafion® suspension from 1-2 to 11. The catalyst inks were prepared from a mixture of the as-prepared samples (25 mg), acetylene black (AB, Strem Chemicals Inc., 5 mg), and 3.33 wt.% K^+ ion-exchanged Nafion® suspension (0.15 mL). The total ink volumes were adjusted to 5 mL by the addition of tetrahydrofuran (THF, Sigma-Aldrich), to give final concentrations of 5 mg sample/mL, 1mg AB/mL, and 1 mg Nafion/mL in the ink. A sample of the ink (3.6 μL) was then drop-casted on a rotating-disk electrode composed of a glassy carbon (GC) disk (0.15 × 0.15 ×π cm^2) (BAS Inc., Japan), which was used as the working electrode after mirror polishing with 0.05 μg alumina slurry (BAS Inc., Japan). The catalyst layer on the GC disk was dried overnight in vacuum at room temperature, and was composed of 0.25 mg oxide /$cm^2{}_{disk}$, 0.05 mg AB /$cm^2{}_{disk}$, and ~0.05 mg Nafion /$cm^2{}_{disk}$.

Electrochemical measurements

Electrochemical measurements were conducted using a rotating ring disk electrode rotator (RRDE-3A, BAS Inc., Japan) at 1600 rpm, in combination with a bipotentiostat (BAS Inc., Japan). In addition, a Pt wire counter electrode, and an Hg/HgO reference electrode (BAS Inc., Japan) filled with 0.10 M KOH (Wako, Ltd., Japan) were used. Electrochemical measurements were conducted with O_2 saturation (30 min bubbling O_2 gas through the solution) at ~20°C, where the equilibrium potential of the O_2/H_2O redox couple was fixed at 0.304V vs. Hg/HgO (equivalent to 1.23 V vs. RHE). During OER current measurements (100 cycles for $Hg_2Ru_2O_7$ and RuO_2, and 50 cycles for $Ca_2Ru_2O_7$ and $Cd_2Ru_2O_7$) for each sample, the potential of the sample-modified GC was controlled from 0.3-0.9 V vs. Hg/HgO (1.226-1.826 V vs. RHE) at 10 mV/s. For all measurements, the OER current density was iR-corrected (R = ~68 Ω) using the measured solution resistance (Supplementary Figure 14), and capacitance-corrected by averaging the anodic and cathodic scans[5] to remove the non-faradaic current contribution. The current densities (mA/$cm^2{}_{disk}$) were obtained by dividing the OER current with the glassy carbon disk electrode area.

Chemical analysis

X-ray photoemission spectra (XPS) were obtained by a XPS-7000 spectrometer (Rigaku Co., Japan) using Al-Kα radiation at room temperature. XPS data were collected with an energy step of 0.1 eV. $Hg_2Ru_2O_7$ and $Ca_2Ru_2O_7$ samples (as-synthesized, as-cast, and after 100 or 50 cycles) were mixed with a Au powder,

which was also used as a Au $4f_{7/2}$ (84.0 eV) standard. XPS data of a RuO_2 powder was collected as a reference for the Ru $3d_{5/2}$ peak. The Ru $3d_{5/2}$ peak positions of $Hg_2Ru_2O_7$ (281.5-281.7 eV) are 0.6-0.8 eV above that of RuO_2 (280.9 eV) for all the samples. This confirms that the Ru of $Hg_2Ru_2O_7$ sustains its Ru^{5+} valence during the OER measurement. The compositional information was obtained by the integrated Hg $4f$ and Ru $3p_{3/2}$ peak areas.

II. Supplementary Results

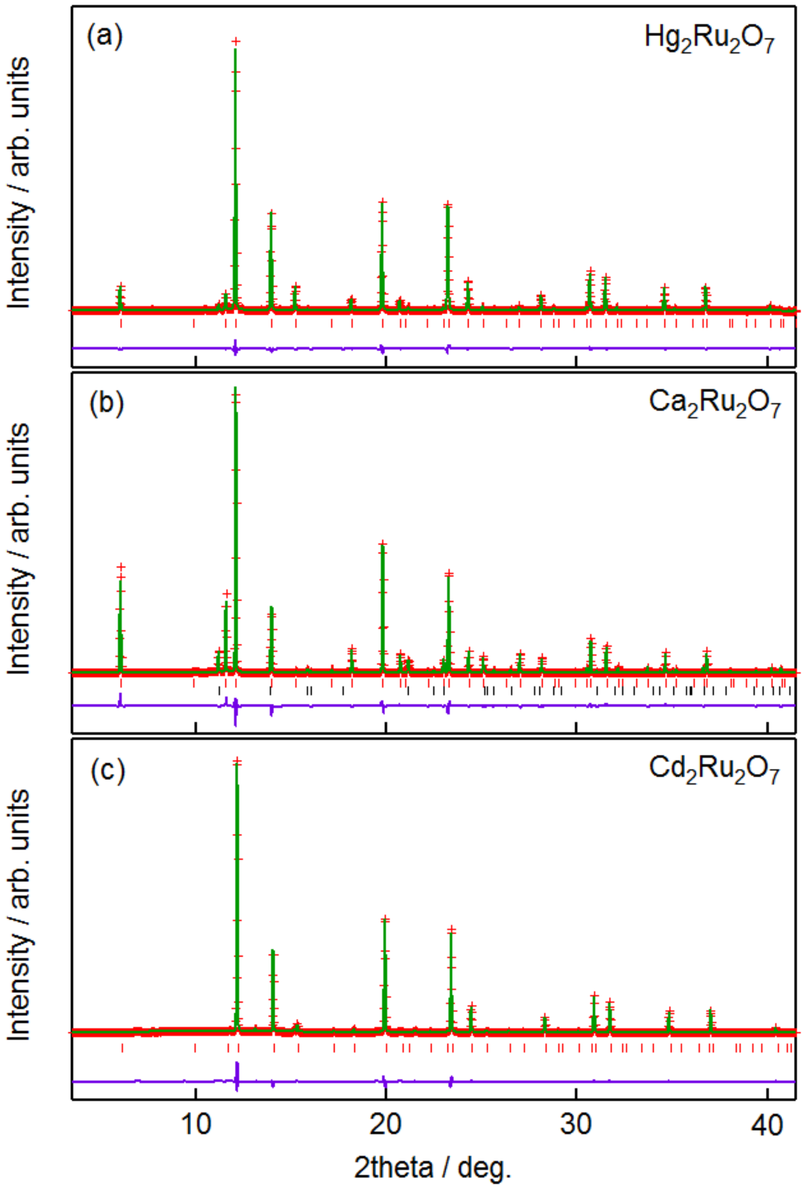

Supplementary Figure 1. Observed synchrotron XRD profiles and Rietveld refinement results for as-synthesized cubic pyrochlore ruthenates. The red cross marks and green lines indicate the observed and calculated profiles, respectively. The red tick marks indicate the Bragg reflection positions of (a) $Hg_2Ru_2O_7$, (b) $Ca_2Ru_2O_7$ and (c) $Cd_2Ru_2O_7$. The black tick marks for (b) $Ca_2Ru_2O_7$ indicate the Bragg reflection positions of the minor secondary phase, RuO_2. The bottom purple curves display the difference between the observed and calculated profiles. Wavelength $\lambda = 0.619925$ Å.

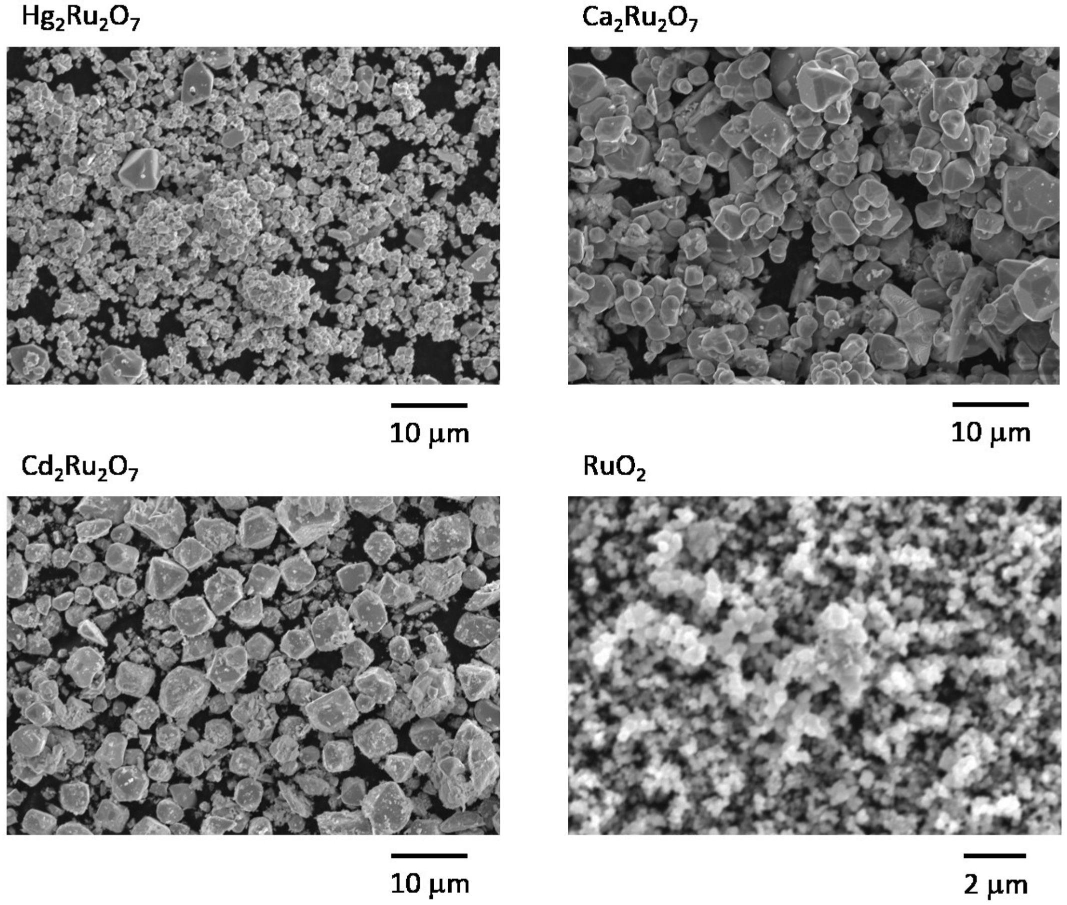

Supplementary Figure 2. SEM images of $Hg_2Ru_2O_7$, $Ca_2Ru_2O_7$, $Cd_2Ru_2O_7$ and RuO_2.

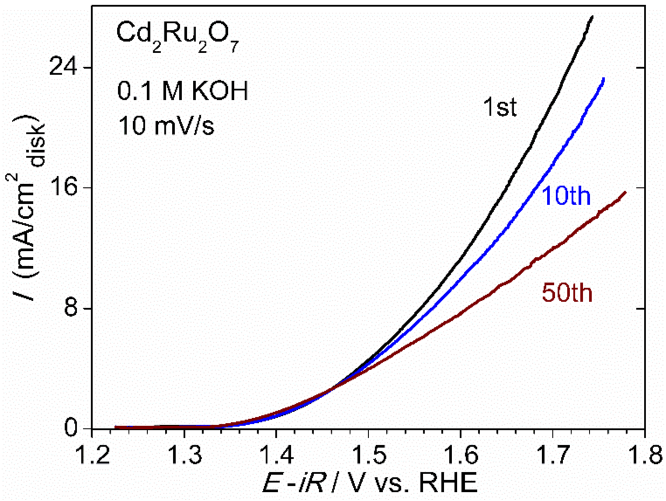

Supplementary Figure 3. The OER catalytic activity of $Cd_2Ru_2O_7$. The linear sweep voltammograms at the scan rate of 10 mV/s for cycle 1, 10 and 50 are shown. The OER measurements were conducted in 0.1 M KOH solution. The current density was normalized to the glassy carbon disk electrode area.

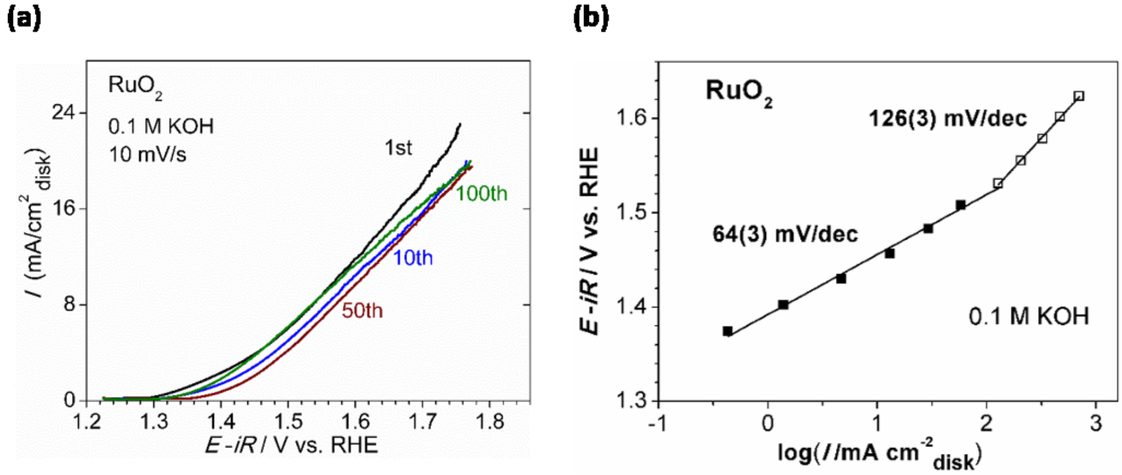

Supplementary Figure 4. The OER catalytic activity of RuO_2. (a) The linear sweep voltammograms (cycle 1, 10, 50 and 100) at the scan rate of 10 mV/s for RuO_2. The OER measurements were conducted in 0.1 M KOH solution. The current density was normalized to the glassy carbon disk electrode area. (b) The OER Tafel plot of RuO_2. The Tafel plot was collected under steady state conditions (constant voltage was applied for 10 min). Standard deviation for the data is within the symbol size.

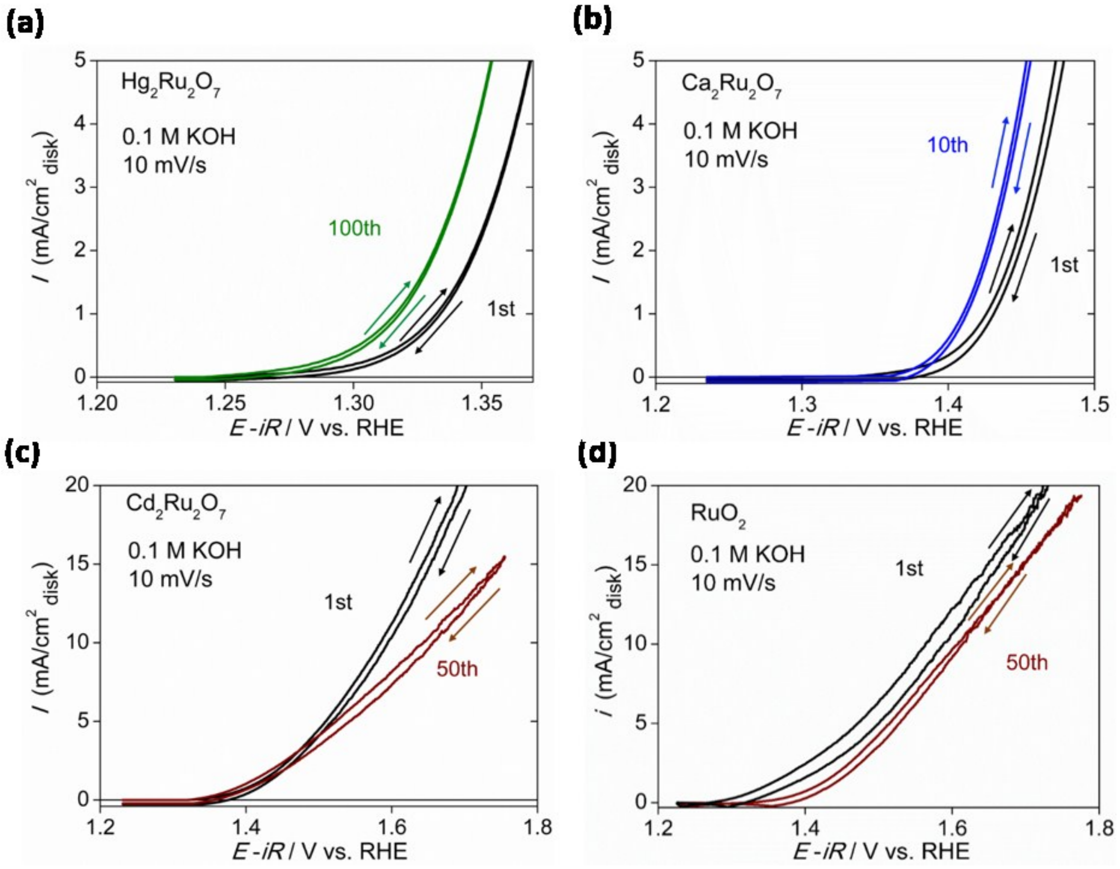

Supplementary Figure 5. The OER catalytic activity of ruthenates. The cyclic voltammograms for (a) $Hg_2Ru_2O_7$ (cycle 1 and 100), (b) $Ca_2Ru_2O_7$ (cycle 1 and 10), (c) $Cd_2Ru_2O_7$ (cycle 1 and 50), and (d) RuO_2 (cycle 1 and 50) at the scan rate of 10 mV/s. The OER measurements were conducted in 0.1 M KOH solutions. The current density was normalized to the glassy carbon disk electrode area. The double-layer capacitance is very small for the catalysts with higher OER activity ($Hg_2Ru_2O_7$and $Ca_2Ru_2O_7$), while it becomes visible in a wider potential scale for $Cd_2Ru_2O_7$and RuO_2. The double-layer capacitance is small and it does not have any clear trend with cycling for the ruthenates since their OER activity stays high over 50 to 100 cycles.

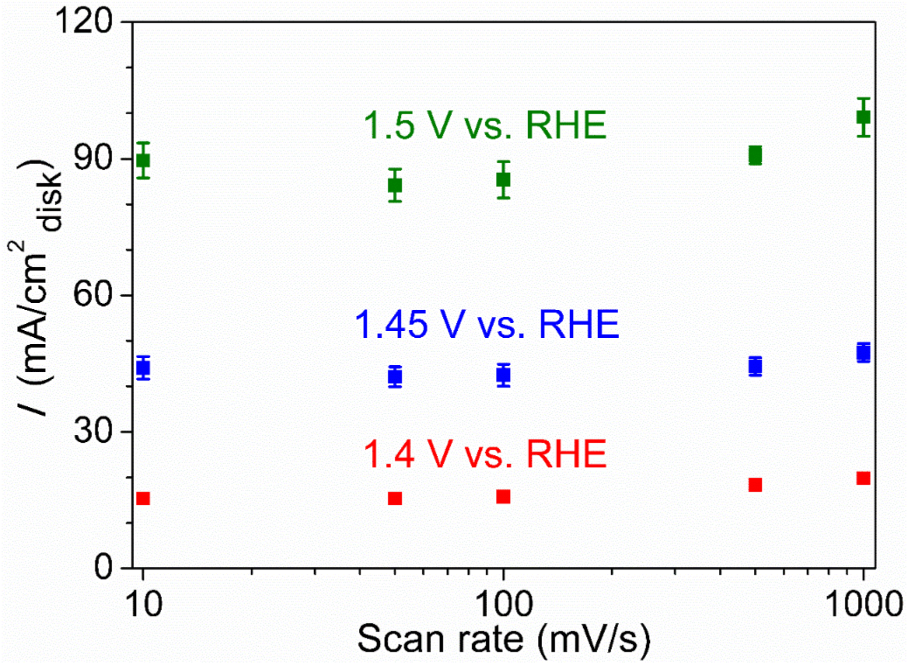

Supplementary Figure 6. Scan rate dependence of the OER current density of $Hg_2Ru_2O_7$. OER activities at 1.4, 1.45, and 1.5 V vs. RHE remain almost constant between the scan rates of 10-1000 mV/s. This implies that the OER measurement for $Hg_2Ru_2O_7$ is not a diffusion-controlled process. The OH^- consumed by the OER is immediately provided by the auto-dissociation of water, which is abundant in 0.1 M KOH aqueous solution The OER measurement is conducted under an electron-transfer controlled process.

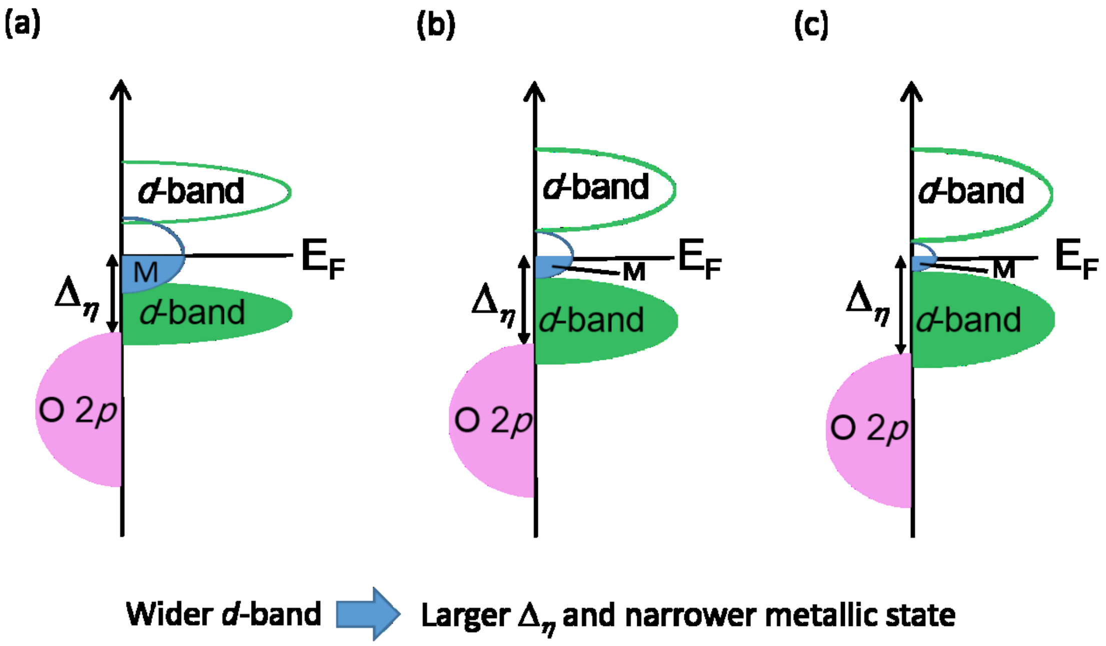

Supplementary Figure 7. Schematic image of the electronic structure in cubic pyrochlore ruthenates. The electronic structure of (a) $Hg_2Ru_2O_7$ at room temperature, (b) $Ca_2Ru_2O_7$ at room temperature, and (c) $Cd_2Ru_2O_7$ at room temperature. Δ_η is a descriptor of the overpotential necessary for the unoccupied *d*-level to overlap with the O *p*-level of the oxygen adsorbates. As stated by Miyazaki *et al.*[33], the metal-insulator transition of $Ca_2Ru_2O_7$ and $Cd_2Ru_2O_7$ is suppressed to a small anomaly at low temperatures (they exhibit metallic conductivity but its temperature dependence shows an insulating behavior) in sharp contrast with $Hg_2Ru_2O_7$. This suggests that the metallic states in $Ca_2Ru_2O_7$ and $Cd_2Ru_2O_7$ are suppressed in comparison with $Hg_2Ru_2O_7$. Since the electronic bands in this figure are composed of Ru 4*d*-electrons (Cd 4*d*-electrons are not responsible for the metal-insulator transition due to the d^{10} closed electron shell with large binding energy) as in $Hg_2Ru_2O_7$ [22], the localized *d*-bands of $Ca_2Ru_2O_7$ and $Cd_2Ru_2O_7$ become wider due to the narrower (or suppressed) metallic states.

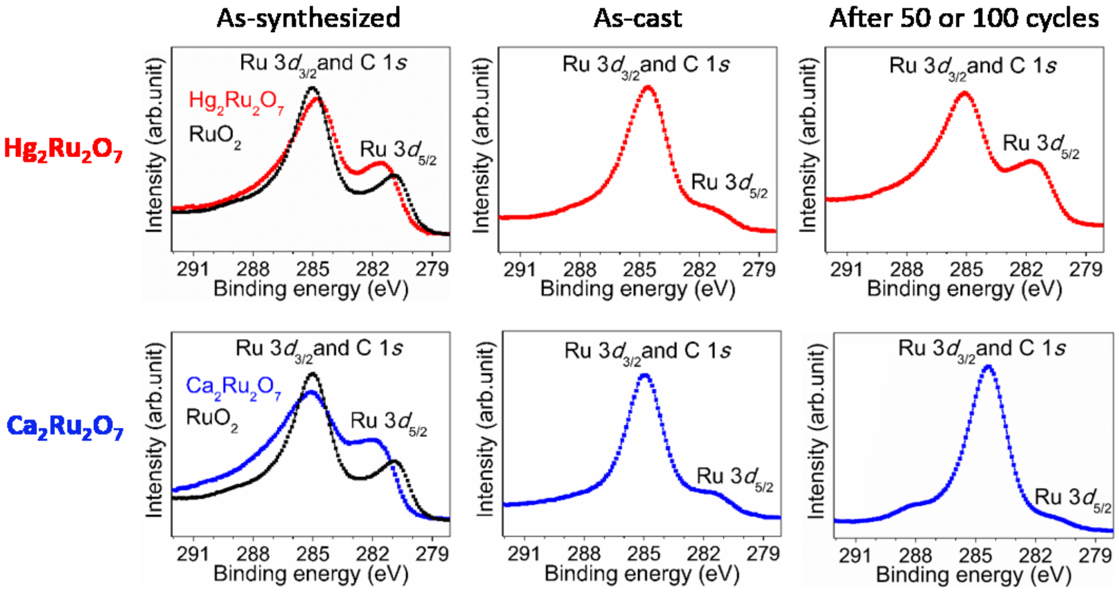

Supplementary Figure 8. X-ray photoemission spectra of Ru 3*d* for $Hg_2Ru_2O_7$ and $Ca_2Ru_2O_7$ catalysts. The Ru 3$d_{5/2}$ peak positions of $Hg_2Ru_2O_7$ are 281.6 eV (as-synthesized), 281.5 eV (as-cast), and 281.7 eV (after 100 cycles of the OER measurement). The Ru 3$d_{5/2}$ peak positions of $Ca_2Ru_2O_7$ are 281.8 eV (as-synthesized), 281.8 eV (as-cast), and 281.4 eV (after 50 cycles of the OER measurement). All of these peak positions are 0.5-0.9 eV above that of the RuO_2 standard (peak position: 280.9 eV, Ru adopts the Ru^{4+} valence). These values are in good agreement with the XPS data for $Hg_2Ru_2O_7$ reported by Yamamoto *et al.*[20]. Therefore, our XPS data confirms that the Ru of the catalysts sustains its Ru^{5+} valence during the OER measurement. The Ru 3*d* peaks were not used for obtaining the compositional information due to the severe overlap of Ru 3$d_{3/2}$ peaks with C 1*s* peaks. The severe overlap of Ru 3$d_{3/2}$ peaks with C 1*s* peaks (the intensity of which greatly depends on the amount of acetylene black attached to the surface of the catalyst) causes the significant change in the intensity ratio between the Ru 3$d_{3/2}$ and 3$d_{5/2}$ peaks for the as synthesized catalyst, the cast catalyst and the catalyst after 50/100 cycles (already at the stage of as synthesized, a small amount of carbon species from the air are attached to the surface).

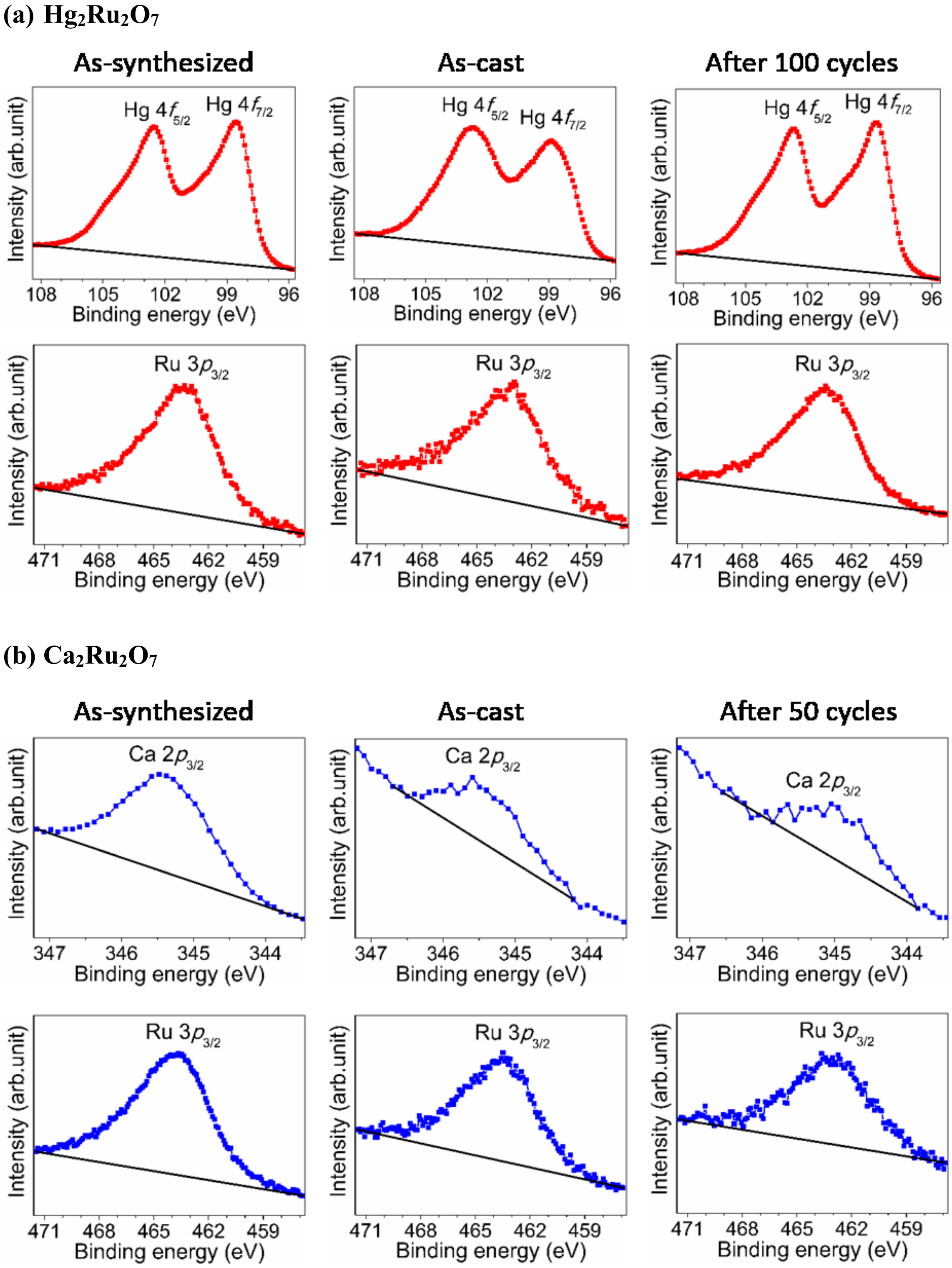

Supplementary Figure 9. X-ray photoemission spectra of Hg 4*f*, Ca 2*p* and Ru 3*p* for $Hg_2Ru_2O_7$ and $Ca_2Ru_2O_7$ catalysts. We obtained the compositional information (see Supplementary Table 4) of $Hg_2Ru_2O_7$ and $Ca_2Ru_2O_7$ catalysts by the integrated peak areas for Hg 4*f*, Ca $2p_{3/2}$ and Ru $3p_{3/2}$.

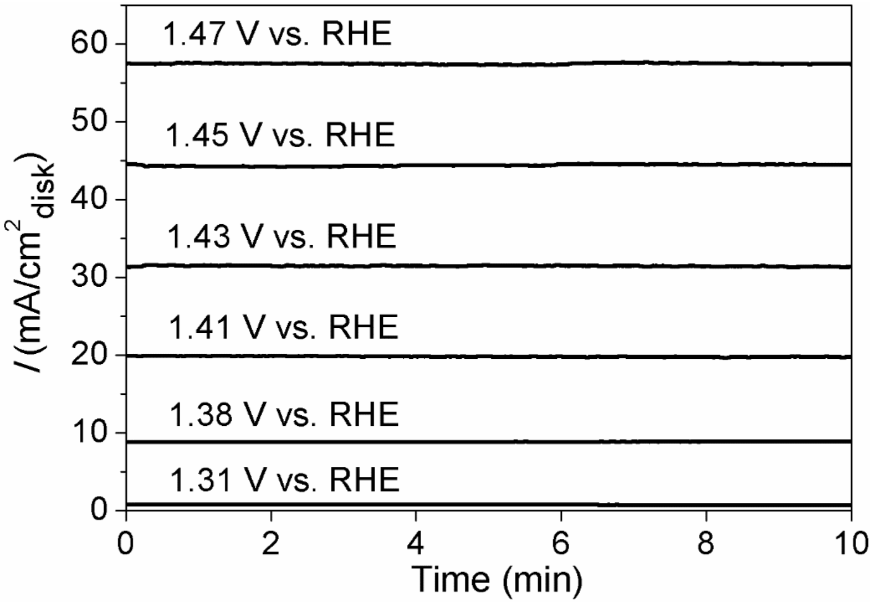

Supplementary Figure 10. Chronoamperograms for $Hg_2Ru_2O_7$ in 0.1 M KOH aqueous solution. Chronoamperograms of $Hg_2Ru_2O_7$ (constant voltage was applied for 10 min) in 0.1 M KOH solution were measured to obtain Tafel plot data under steady state conditions. Chronoamperogram at 1.31 V vs. RHE shows that the onset potential of $Hg_2Ru_2O_7$ is below 1.31 V vs. RHE.

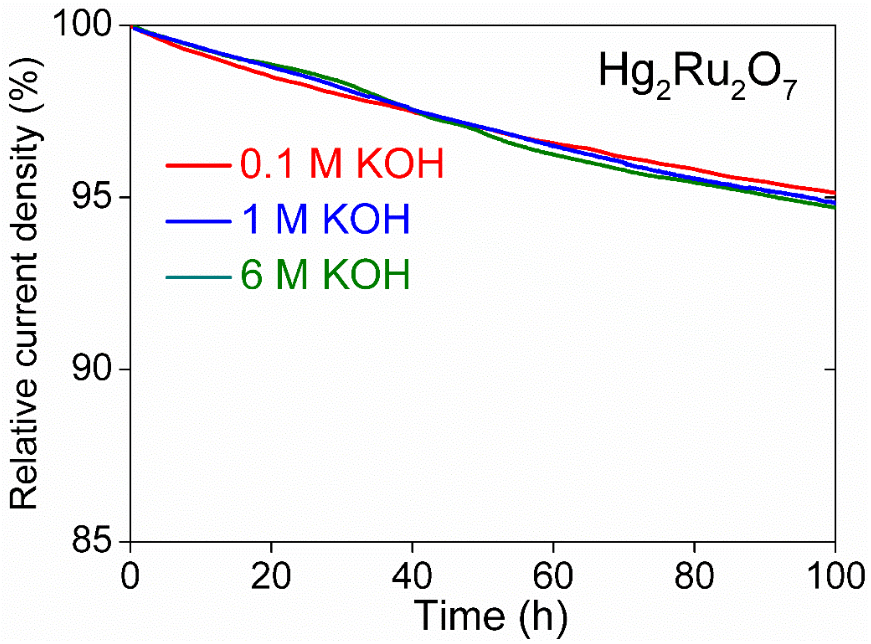

Supplementary Figure 11. OER catalytic stability of $Hg_2Ru_2O_7$ in KOH solutions. Chronoamperometric curves (time dependence of relative current density) at 1.5 V vs. RHE (without *iR*-correction) for $Hg_2Ru_2O_7$ in 0.1 M, 1 M and 6 M KOH solutions.

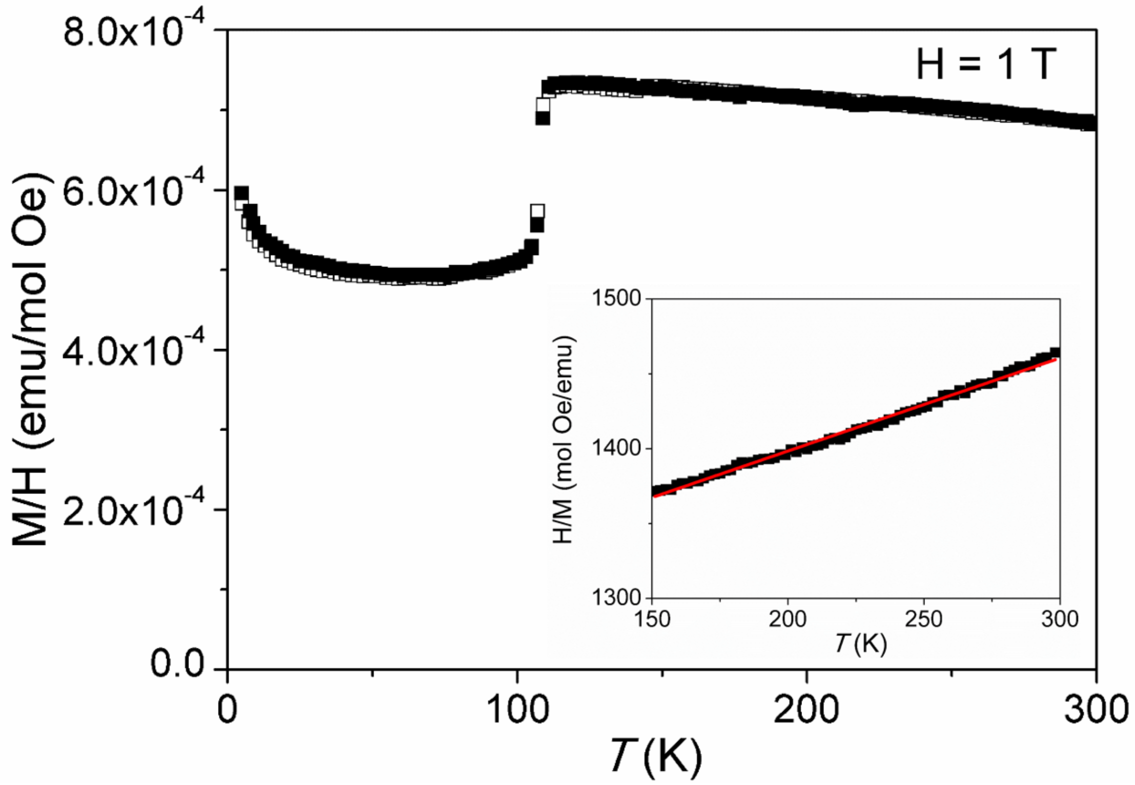

Supplementary Figure 12. Zero field-cooled and field-cooled (open and closed symbols) magnetic susceptibility (M/H) curves for $Hg_2Ru_2O_7$ in a field of 1 T. The inset shows the Curie-Weiss fit to inverse susceptibility between 150 and 300 K. The Curie-Weiss fit gives an effective moment (μ_{eff}) of ~3.6 μ_B and a Weiss temperature (θ) of ~2100 K. The unphysical value of θ suggests that $Hg_2Ru_2O_7$ adopts an intermediate state of paramagnetism between Curie-Weiss paramagnetism and Pauli paramagnetism, which was also proposed by Yamamoto *et al.*[20] The magnetic susceptibility curves were measured using a superconducting quantum interference device magnetometer (Quantum Design, VSM) between 5 and 300 K.

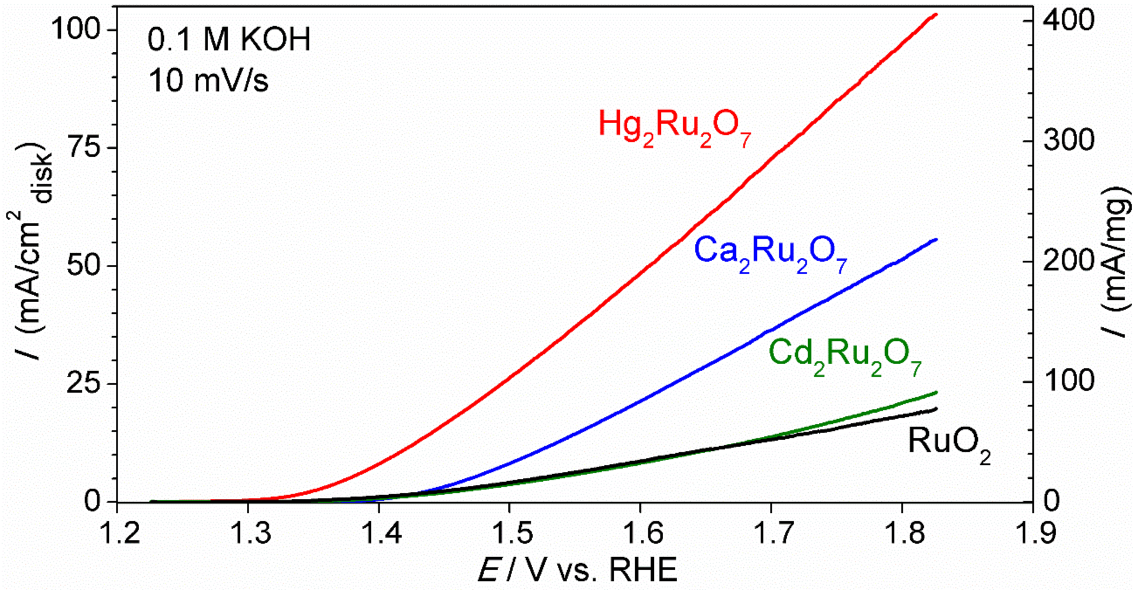

Supplementary Figure 13. OER catalytic activity of the cubic pyrochlore ruthenates. Linear sweep voltammograms without *iR*-correction for $Hg_2Ru_2O_7$, $Ca_2Ru_2O_7$, $Cd_2Ru_2O_7$, and RuO_2.

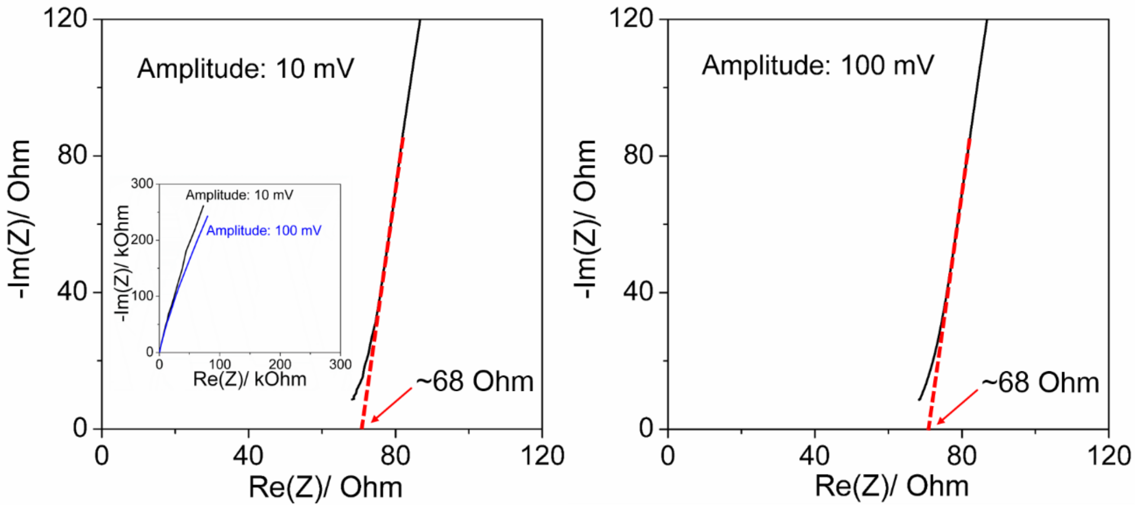

Supplementary Figure 14. Nyquist plots of impedance spectra of the OER measurement system in this study are shown (expanded view between the frequency of 40 kHz and 500 Hz). The solution resistance of the OER measurement system was estimated as ~68 Ω by the extrapolation of the Nyquist plots at 10 mV and 100 mV amplitude around the open circuit potential (-0.03 to 5mV vs. Hg/HgO). The inset shows the overall view of the impedance spectra between the frequency of 40 kHz and 100 mHz. Impedance spectra were measured using a potentiostat/galvanostat with frequency response analyzer (Bio-Logic SAS, SP-150).

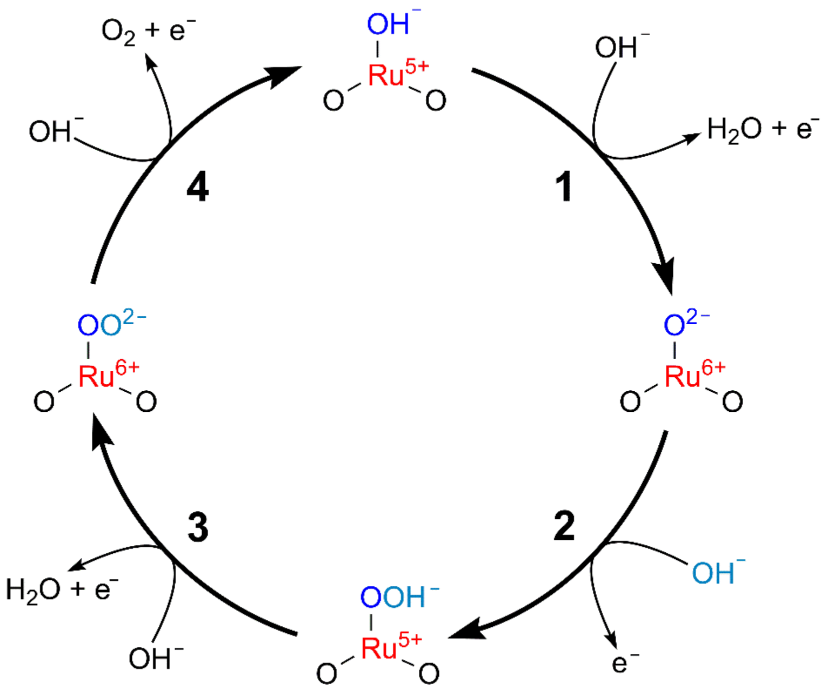

Supplementary Figure 15. The OER mechanism of cubic pyrochlore ruthenates in this study based on the electron transfer between Ru ion and the OH^- adsorbate. The rate-determining step is either the formation of O-O bond (reaction 2) or the deprotonation (reaction 3) along with the redox reaction of Ru ions in analogy with the perovskite oxides in Suntivich *et al.*[5] and Yagi *et al.*[6], and the high OER activity of $Hg_2Ru_2O_7$ (especially the low overpotential) can be explained by the smooth electron transfer between Ru ion and the OH^- adsorbate.

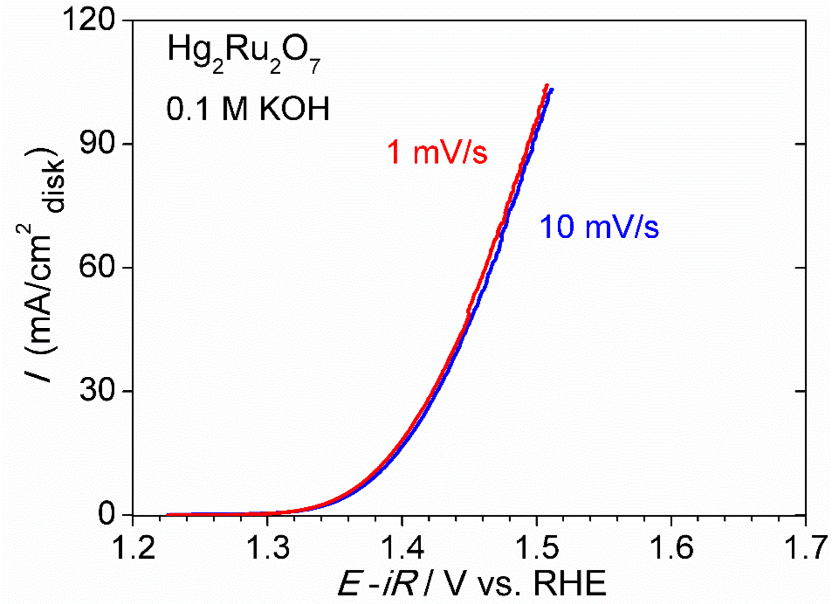

Supplementary Figure 16. The linear sweep voltammogram of $Hg_2Ru_2O_7$ at the scan rate of 1 mV/s. The voltammogram at the scan rate of 10 mV/s is shown as a reference. The OER measurements were conducted in 0.1 M KOH solution.

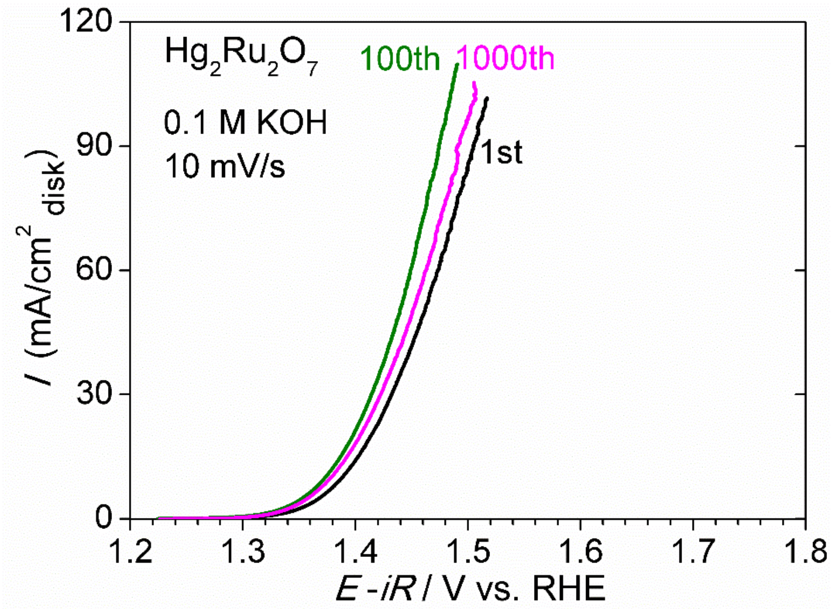

Supplementary Figure 17. The linear sweep voltammogram of $Hg_2Ru_2O_7$ after 1000 OER cycles. The voltammograms of cycle 1 and cycle 100 are shown as references. The OER measurements were conducted in 0.1 M KOH solution.

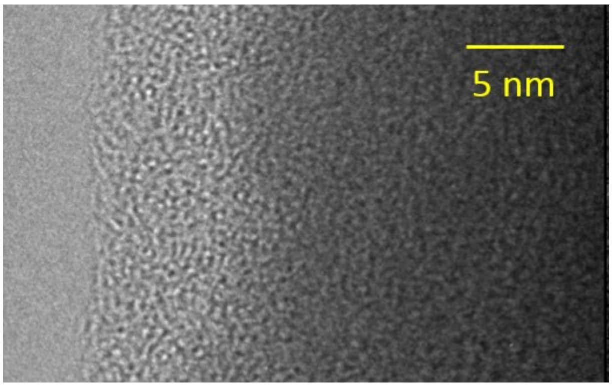

Supplementary Figure 18. Expanded view of the HRTEM image for $Ca_2Ru_2O_7$ after 50 OER cycles. The surface of $Ca_2Ru_2O_7$ was covered with amorphous layer within the detectable limit (~25 nm) of the large grains of $Ca_2Ru_2O_7$.

Supplementary Table 1. Crystallographic results for cubic pyrochlore ruthenates. These results were obtained by conducting Rietveld refinement on the synchrotron XRD profiles**.** All these samples adopt the space group of *Fd-3m*. Atomic positions are Hg/Ca/Cd (0.5, 0.5, 0.5), Ru (0, 0, 0), O1 (*x*, 1/8, 1/8) and O2 (3/8, 3/8, 3/8). Lattice constants, cell volume, atomic position *x* of O1, isotropic temperature factor B_{eq}, Ru-Ru, Ru-O, O-O distances, R-O-R, O-R-O angles, R_{wp}, and goodness-of-fit (GOF) with a standard deviation.

	$Hg_2Ru_2O_7$	$Ca_2Ru_2O_7$	$Cd_2Ru_2O_7$
a (Å)	10.207(3)	10.194(2)	10.121(2)
V (Å^3)	1063.5(9)	1059.8(7)	1036.6(8)
Hg/Ca/Cd _B_{eq}	1.45(2)	4.01(5)	1.79(4)
Ru_ B_{eq}	1.01(3)	1.39(2)	1.48(5)
O1_*x*	0.3161(5)	0.3190(3)	0.3201(6)
O1_ B_{eq}	1.1(2)	1.81(8)	1.3(1)
O2_ B_{eq}	1.1(2)	1.81(8)	1.3(1)
Ru-Ru (Å)	3.6087(8)	3.6041(6)	3.5783(6)
Ru-O (Å)	1.929(2)	1.934(1)	1.925(2)
O-O (Å)	2.759(6) / 2.690(2)	2.797(4) / 2.673(1)	2.793(7) / 2.679(2)
Ru-O-Ru (deg.)	139.0(3)	137.4(3)	136.7(3)
O-Ru-O (deg.)	88.6(2)	87.4(1)	87.0(2)
R_{wp}	0.0674	0.0931	0.0965
GOF	1.10	1.81	1.55

Supplementary Table 2. BET Surface area of $Hg_2Ru_2O_7$, $Ca_2Ru_2O_7$, $Cd_2Ru_2O_7$ and RuO_2. The specific surface area A_s was determined by nitrogen BET surface area measurements at 77 K using a conventional high vacuum static system. Since the BET surface areas of the ruthenates are somewhat similar (all of the ruthenates are low surface area catalysts), the current density normalized to the disk electrode area does not bias the comparison of the catalytic activity.

Material	A_s (m^2/g)
$Hg_2Ru_2O_7$	0.94(4)
$Ca_2Ru_2O_7$	0.93(4)
$Cd_2Ru_2O_7$	1.84(1)
RuO_2	7.43(12)

Supplementary Table 3. Ionicity of bonds for cubic pyrochlore ruthenates. The ionicity is used as an indicator of the cation-oxygen bond covalency in $Hg_2Ru_2O_7$, $Ca_2Ru_2O_7$ and, $Cd_2Ru_2O_7$. The ionicity of cation-oxygen bonds was calculated using the equation by Pauling *et al.*[S2], f_i = 1- exp(- $\Delta\chi^2/4$), where f_i and $\Delta\chi$ denote bond ionicity and Pauling's electronegativity difference between the cation-anion pair, respectively. This table demonstrates that Hg-O and Ru-O bonds are covalent (ionicity < 50 %), while Ca-O and Cd-O bonds are ionic (ionicity > 50 %).

Bond	$\Delta\chi$	Bond ionicity (%)
Hg-O	1.44	40.5
Ru-O	1.24	31.9
Ca-O	2.44	77.4
Cd-O	1.75	53.5

Supplementary Table 4. Compositional information of $Hg_2Ru_2O_7$ and $Ca_2Ru_2O_7$ catalysts. The elemental ratios (Hg/Ru and Ca/Ru) of the catalysts before and after the OER measurements were determined by X-ray photoemission spectra (XPS). The Hg/Ru ratio was obtained by the integrated Hg 4*f* and Ru $3p_{3/2}$ peak areas in comparison with the as-synthesized $Hg_2Ru_2O_7$ (Supplementary Figure 9(a)). The Ca/Ru ratio was obtained by the integrated Ca $2p_{3/2}$ and Ru $3p_{3/2}$ peak areas in comparison with the as-synthesized $Ca_2Ru_2O_7$ (Supplementary Figure 9(b)). Only 8 % of the Hg^{2+} in $Hg_2Ru_2O_7$ dissolved in the alkaline solution during the OER measurement, while 25 % of the Ca^{2+} in $Ca_2Ru_2O_7$ dissolved in the alkaline solution even at the stage of as-cast. The almost constant Ca/Ru ratio during the OER measurement can be explained by the diffusion of Ca^{2+}at the boundary of amorphous and crystalline layer due to the thick amorphous layer over 25 nm.

Catalysts	Hg/Ru or Ca/Ru (%)
$Hg_2Ru_2O_7$ as-cast	100
$Hg_2Ru_2O_7$ after 100 cycles	92
$Ca_2Ru_2O_7$ as-cast	75
$Ca_2Ru_2O_7$ after 50 cycles	73

Supplementary Table 5: OER overpotentials of cubic pyrochlore ruthenates and the highly active chalcogenide based OER catalysts. These catalysts were deposited on a glassy carbon disk electrode and the OER measurements were conducted in KOH solutions. The overpotentials of chalcogenide OER catalysts were taken from Swesi *et al.*[S3]. $Hg_2Ru_2O_7$ (non-Fermi liquid) exhibits a comparable overpotential with the highly active chalcogenide OER catalysts.

Material	Overpotential η (V)
$Hg_2Ru_2O_7$	0.148(7)
$Ca_2Ru_2O_7$	0.279(28)
$Cd_2Ru_2O_7$	0.380(32)
Ni_3S_2–Ni foam	0.187
Ni_3Se_2–Ni foam	0.27(20)
Ni_3Se_2	0.31(20)

Supplementary Table 6: The OER performance of the Ru-based catalysts in KOH solutions. The data of $Pb_2Ru_2O_{6.5}$ [30], $Bi_{2.4}Ru_{1.6}O_7$ [30], $CaRuO_3$ [S4], $SrRuO_3$ [S5], and RuO_2-NiO [S6] were taken from the previous literature. The turnover frequency (TOF) was calculated by TOF = $j/4Fn$, where j is the OER current at 1.5 V vs. RHE, F is the Faraday constant, and n is the number of moles of the Ru atom on the working electrode. $Hg_2Ru_2O_7$ (non-Fermi liquid) exhibits the highest OER activity in Ru-based catalysts.

Material	Overpotential η (V)	TOF (s^{-1})
$Hg_2Ru_2O_7$	0.148(7)	0.376
$Ca_2Ru_2O_7$	0.279(28)	0.0268
$Cd_2Ru_2O_7$	0.380(32)	0.0119
$Pb_2Ru_2O_{6.5}$	0.20	0.187
$Bi_{2.4}Ru_{1.6}O_7$	0.38	0.0126
$CaRuO_3$	0.21	-
$SrRuO_3$	-	4.87×10^{-4}
RuO_2-NiO	0.31	-
RuO_2	0.349(18)	6.60×10^{-3}

Supplementary References

[S1] A. A. Coelho, TOPAS-Academic version 5. *Bruker AXS GmbH*, Karlsruhe, Germany, **2012**.
[S2] L. Pauling, The Nature of the Chemical Bond. *Cornell University Press*, Ithaca, NY, **1960**.
[S3] A. T. Swesi, J. Masud, M. Nath, *Energy Environ. Sci.* **2016**, *9*, 1771.
[S4] E. O. Chi, Y. U. Kwon, S. Mho, *Bull. Korean Chem. Soc.* **1997**, *18*, 972.
[S5] B. J. Kim, D. F. Abbott, X. Cheng, E. Fabbri, M. Nachtegaal, F. Bozza, I. E. Castelli, D. Lebedev, R. Schäublin, C. Copéret, T. Graule, N. Marzari, T. J. Schmidt, *ACS Catal.* **2017**, *7*, 3245.
[S6] K. Juodkazis, J. Juodkazytė, R. Vilkauskaitė, B. Šebeka, V. Jasulaitienė, *Chemija* **2008**, *19*, 1.
